# The revival of the Gini importance?

**DOI:** 10.1093/bioinformatics/bty373

**Published:** 2018-05-10

**Authors:** Stefano Nembrini, Inke R König, Marvin N Wright

**Affiliations:** 1 Department of Epidemiology, College of Public Health and Health Professions & College of Medicine, University of Florida, Gainesville, FL, USA; 2 Institut für Medizinische Biometrie und Statistik, Universität zu Lübeck, Universitätsklinikum Schleswig-Holstein, Campus Lübeck, Lübeck, Germany; 3 Leibniz Institute for Prevention Research and Epidemiology – BIPS, Bremen, Germany

## Abstract

**Motivation:**

Random forests are fast, flexible and represent a robust approach to analyze high dimensional data. A key advantage over alternative machine learning algorithms are variable importance measures, which can be used to identify relevant features or perform variable selection. Measures based on the impurity reduction of splits, such as the Gini importance, are popular because they are simple and fast to compute. However, they are biased in favor of variables with many possible split points and high minor allele frequency.

**Results:**

We set up a fast approach to debias impurity-based variable importance measures for classification, regression and survival forests. We show that it creates a variable importance measure which is unbiased with regard to the number of categories and minor allele frequency and almost as fast as the standard impurity importance. As a result, it is now possible to compute reliable importance estimates without the extra computing cost of permutations. Further, we combine the importance measure with a fast testing procedure, producing *p*-values for variable importance with almost no computational overhead to the creation of the random forest. Applications to gene expression and genome-wide association data show that the proposed method is powerful and computationally efficient.

**Availability and implementation:**

The procedure is included in the ranger package, available at https://cran.r-project.org/package=ranger and https://github.com/imbs-hl/ranger.

**Supplementary information:**

Supplementary data are available at *Bioinformatics* online.

## 1 Introduction

Random forests (RFs, Breiman, 2001) are a popular machine learning approach to analyze high dimensional data from the life sciences such as gene expression (Díaz-Uriarte and Alvarez de Andrés, 2006) or genome-wide association studies (GWAS) (Goldstein *et al.*, 2011). An important reason for their popularity is the availability of variable importance measures (VIMs). The most widely used VIMs are the impurity importance and the permutation importance (Breiman, 2001). The impurity importance is also known as the mean decrease of impurity (MDI), the permutation importance as mean decrease of accuracy (MDA), see Sections 2.2 and 2.3 for further details. Since the Gini index is commonly used as the splitting criterion in classification trees, the corresponding impurity importance is often called Gini importance. The impurity importance is known to be biased in favor of variables with many possible split points, i.e. categorical variables with many categories or continuous variables (Breiman *et al.*, 1984; Strobl *et al.*, 2007), and in favor of variables with high category frequencies (Nicodemus, 2011), e.g. single-nucleotide polymorphisms (SNPs) with high minor allele frequencies (MAF) in GWAS (Boulesteix *et al.*, 2012). The permutation importance does not suffer from these kinds of bias and is consequently generally preferred (Nicodemus *et al.*, 2010; Szymczak *et al.*, 2016; Ziegler and König, 2014). On the other hand, for high dimensional data, the permutation importance is very computationally intensive and Calle and Urrea (2011) showed that rankings based on the impurity VIM can be more robust to perturbations of the data compared with those obtained with the permutation importance.

VIMs can be used to identify relevant features or perform variable selection. Variable selection approaches can be divided into *performance-based approaches* and *test-based approaches* (Hapfelmeier and Ulm, 2013). The former include, for instance, the method proposed by Díaz-Uriarte and Alvarez de Andrés (2006), which ranks variables with respect to their association with a disease or trait through a VIM and recursively removes a certain number or percentage of these ranked variables. The latter include statistical testing procedures for VIMs which select variables at the *p*-value scale and keep only significantly associated variables (Altmann *et al.*, 2010; Janitza *et al.*, 2016; Kursa *et al.*, 2010).

Here we focus on a class of test-based approaches that are reminiscent of the methods explained in Tuv *et al.* (2006) and Rudnicki *et al.* (2006), which use permutations of the original variables, either by permuting the rows or the columns of the original data in order to distinguish important from unimportant variables. In a similar fashion, Sandri and Zuccolotto (2008) added so-called pseudo variables to a dataset, based on the idea of Wu *et al.* (2007). These pseudo variables are permuted versions of the original variables, which can be used to correct for bias. Kursa *et al.* (2010) also used pseudo variables (called *shadow attributes*) to distinguish important from unimportant variables. However, all these approaches are based on several RF runs, including computation of the VIMs in every run, which can be computationally slow for high dimensional datasets (Degenhardt *et al.*, 2017). In the same line, Walters *et al.* (2012) found the approach of Sandri and Zuccolotto (2008) to be infeasible for large datasets because of the computational burden and proposed a modified approach to reduce the bias with regard to MAF in GWAS. However, the modified approach does not completely eliminate the bias.

Building upon the method of Sandri and Zuccolotto (2008), we present a debiased VIM for RF, which is as unbiased as the permutation importance proposed in Janitza *et al.* (2016) and is almost as computationally fast as the impurity importance. Specifically, we achieve this by computing the corrected impurity importance inside a single RF run and by avoiding the computing cost of permutations. In simulation studies we compare the distribution and the bias of different importance measures and show that the proposed method is unbiased regarding the number of categories and the MAF. Because of its symmetry property (see Section 3.1.1), we further combine it with the testing procedure of Janitza *et al.* (2016), producing *p*-values for variable importance with almost no computational overhead to the creation of the RF.

## 2 Materials and methods

### 2.1 Random forests

RFs (Breiman, 2001) are ensembles of classification, regression or survival trees. As in bagging (Breiman, 1996), each tree is grown on a bootstrap sample of the original dataset. Alternatively, the trees might be grown on subsamples (subagging). As a second component of randomization, only a random subset of the variables is considered as splitting candidates at each split in the trees. Splitting rules in RF maximize the decrease of impurity that is introduced by a split. For classification, the impurity reduction is typically measured by the Gini index (Breiman *et al.*, 1984), for regression by the sum of squares (Ishwaran, 2015) and for survival outcomes by the log-rank statistic (Ishwaran *et al.*, 2008) or Harrell’s C-index (Schmid *et al.*, 2016). After growing a prespecified number of trees in the ensemble, the results of the trees are aggregated, where the form of aggregation depends on the outcome type.

Besides the number of trees, the most important parameters are the size of the random subsets of variables considered for splitting (the so-called mtry value) and the size of the trees. In most implementations, the default value for mtry is $\sqrt{p}$ for classification and survival forests and p/3 for regression forests, where *p* is the number of variables in the dataset. However, for datasets with many noisy variables, e.g. in genomics, a larger value might be required (Goldstein *et al.*, 2011). The tree size is usually controlled by a prespecified terminal node size or tree depth. For classification outcomes, the trees are typically grown to purity instead, corresponding to a terminal node size of 1. To achieve the best prediction accuracy without overfitting, these parameters should be tuned, e.g. by a nested cross validation.

As a positive side effect of the bagging or subagging, every tree in the ensemble has a set of observations, which were not used to grow the tree. These are termed out-of-bag (OOB) observations, and they can be used to estimate the prediction accuracy or variable importance, as described in the next section.

### 2.2 Permutation importance

In the permutation variable importance (Breiman, 2001), a variable is identified as important if it has a positive effect on the prediction performance, estimated by the OOB prediction error. To calculate the permutation importance of the variable *X_i_*, its original association with the response *Y* is broken by randomly permuting the values of all individuals for *X_i_*. With this permuted data, the tree-wise OOB estimate of the prediction error is computed. The difference between this estimate and the OOB error without permutation, averaged over all trees, is the permutation importance of the variable *X_i_*. This procedures is repeated for all variables of interest $X_{1},\ldots ,X_{p}$. The larger the permutation importance of a variable, the more relevant the variable is for the overall prediction accuracy. For a detailed description, see, e.g. Breiman (2001).

Because the distribution of the permutation importance is not perfectly symmetric around zero for unassociated variables, Janitza *et al.* (2016) proposed a modified version, termed holdout importance. The idea is to compute the variable importance with a 2-fold cross validation, i.e. split the data in two halves, grow an RF on each half and compute permutation importance on the other half instead of the OOB observations of each tree. The procedure is available in the ranger package (Wright and Ziegler, 2017) by the function holdoutRF().

### 2.3 Impurity importance

As described above, RF splitting rules maximize the impurity reduction introduced by a split. For the impurity importance, a split with a large decrease of impurity is considered important and as a consequence variables used for splitting at important splits are also considered important. Based on this idea, the impurity importance for a variable *X_i_* is computed by the sum of all impurity decrease measures of all nodes in the forest at which a split on *X_i_* has been conducted, normalized by the number of trees. For classification, the impurity is usually measured by the Gini impurity:

$$\hat{\Gamma }(t)=\sum_{j=1}^{J}{\hat{\phi }}_{j}(t)(1-{\hat{\phi }}_{j}(t)),$$

where ${\hat{\phi }}_{j}(t)$ is the class frequency for class *j* in the node *t* (Ishwaran, 2015). The decrease of impurity is the difference between a node’s impurity and the weighted sum of the impurity measures of the two child nodes (the Gini index). For regression, the same procedure with the sum of squares as impurity measure is used. Using the variance as impurity measures leads to the same results except for a constant factor, see Ishwaran (2015) for details.

The split variable selection in RF is known to be biased in favor of variables with many possible split points (Breiman *et al.*, 1984; Wright *et al.*, 2017). The reason is that—just by chance—a variable with many possible split points will have a higher probability to produce a split with a large impurity reduction than a variable with fewer possible split points, even if both variables are unassociated with the outcome; see Wright *et al.* (2017) for a detailed explanation. Since the impurity importance is calculated based on the variables selected for splitting, it is biased as well (Strobl *et al.*, 2007). For example, continuous variables will receive higher impurity importance values than dichotomous variables, even if both are uninformative. The problem intensifies if all 2-partitions are considered when splitting nominal factors, as it is usually done. Furthermore, even within variables with the same number of categories, variables with high entropy, i.e. variables with high category frequencies (Nicodemus, 2011) and SNPs with high MAF (Boulesteix *et al.*, 2012) are systematically preferred.

### 2.4 Actual impurity reduction importance

Following the definitions of Sandri and Zuccolotto (2008), the impurity importance ${\text{VIM}}_{X_{i}}$ can be expressed as a sum of two components:

$${\text{VIM}}_{X_{i}}=\Delta H_{Y}\left(X_{i}\right)+\Delta b_{Y}\left(X_{i}\right)$$

where $\Delta H_{Y}\left(X_{i}\right)$ is the part of heterogeneity reduction directly related to the true importance of *X_i_* and $\Delta b_{Y}\left(X_{i}\right)$ is the part of heterogeneity reduction solely due to the structure of *X_i_*, i.e. a positive bias. The aim of an unbiased impurity importance measure is to quantify the amount of only the heterogeneity reduction directly related to the true importance $\Delta H_{Y}\left(X_{i}\right)$, i.e. the actual impurity reduction.

Sandri and Zuccolotto (2008) proposed to augment the original dataset {Y;X} with pseudo data ***Z***, which is uninformative but shares the structure of ***X***. For each original variable *X_i_*, a pseudo variable *Z_i_* is added to the dataset. The impurity importance of these pseudo variables is:

$${\text{VIM}}_{Z_{i}}=\Delta H_{Y}\left(Z_{i}\right)+\Delta b_{Y}\left(Z_{i}\right).$$

By construction of *Z_i_*, the heterogeneity reduction directly related to its true importance is equal to 0, which means that the importance of *Z_i_* contains bias only, i.e.

$${\text{VIM}}_{Z_{i}}=\Delta b_{Y}\left(Z_{i}\right).$$

Since *Z_i_* shares the structure of *X_i_*, the variable and its pseudo variable have the same amount of bias, leading to:

$${\text{VIM}}_{Z_{i}}=\Delta b_{Y}\left(X_{i}\right).$$

Thus, their bias-corrected impurity importance for *X_i_* can be computed as:

$$\hat{{\text{VIM}}_{X_{i}}^{*}}=R^{-1}\sum_{r=1}^{R}\left(\hat{{\text{VIM}}_{X_{i}}}-\hat{{\text{VIM}}_{Z_{i}}}\right)$$

Sandri and Zuccolotto (2008) create the pseudo variables ***Z*** by permuting the rows of ***X***. For each forest *r*, a permuted version of ***X*** is added to the dataset, and the process is replicated *R* times (they use *R* = 300 for real-life data), and the results are averaged.

However, doubling the size of the dataset from *n *×* p* to n×2p decreases the computational performance substantially and increases memory requirements for the analysis of large datasets. The use of replications further increases the computation time. As a result, it has been argued that this renders the approach infeasible for GWAS (Walters *et al.*, 2012).

We propose a modified approach of that by Sandri and Zuccolotto (2008). Let us recall that a linear reordering π of a given variable *X_i_* is a bijective function which defines the rearrangement of its elements {1,…,n} so that the resulting reordered variable can be defined as _π_*X_i_* (Bóna, 2012). Before training the RF, a single random reordering π of the sample ID’s is drawn.

In a regular RF, at each tree node, mtry splitting candidate variables are sampled from O≡{1,…,p}. Instead, we sample from 1 to 2*p*, i.e. variables are sampled from O∪ℙ, where ℙ≡{p+1,…,2p}. If the chosen variable index j∈O, then $X_{i=j}$ is used for splitting as usual. If j∈ℙ, the variable _π_*X_i=j−p_*, i.e. the original variable with reordered sample ID’s, is used for splitting. If j∈O, the impurity reduction by this split contributes normally to the variable importance of *X_i_*, whereas it is subtracted if j∈ℙ. Note that no replications of the procedure are performed.

Finally, after the whole forest has been grown, the estimated debiased impurity importance of a variable *X_i_* is computed as:

$${\hat{\text{AIR}}}_{X_{i}}={\hat{\text{VIM}}}_{\overset{j\in O}{X_{i}}}-{\hat{\text{VIM}}}_{\overset{j\in \mathbb{P}}{X_{i}}}.$$

We refer to this new VIM as actual impurity reduction (AIR), to avoid possible misunderstandings with other VIMs. The AIR importance is now available in the ranger package (Wright and Ziegler, 2017).

### 2.5 Importance testing procedures

In order to select relevant variables, a usual approach is to select a fixed number or percentage of the highest ranking variables. However, a more appropriate procedure would be to perform statistical testing and select all variables significantly associated with the outcome.

Given an unbiased VIM (${\text{VIM}}_{X_{i}}^{\prime }$), we want to test the null hypothesis:

$$H_{0}:{\text{VIM}}_{X_{i}}^{\prime }\leq 0$$

against the alternative

$$H_{1}:{\text{VIM}}_{X_{i}}^{\prime }>0.$$

A straightforward approach is to use a permutation test, as proposed by Altmann *et al.* (2010) and Hapfelmeier and Ulm (2013). These approaches work well in low-dimensional settings. However, a new RF has to be grown for every permutation, making them infeasible for high dimensional data as in genomics.

An alternative approach for high dimensional data has been proposed by Janitza *et al.* (2016). The algorithm works as follows:

1. Grow a RF on the data {Y,X} and compute an unbiased variable measure $\hat{{\text{VIM}}_{X_{i}}^{\prime }}$ for each variable *X_i_*
2. Approximate the null distribution *M* by mirroring the empirical distribution of the observed negative and zero variable importance scores as $M=M_{1}\cup M_{2}\cup M_{3}$, where
  $$M_{1}=\left\{\hat{{\text{VIM}}_{X_{i}}^{\prime }}|\hat{{\text{VIM}}_{X_{i}}^{\prime }}<0;\;i=1,\ldots ,p\right\},$$
  $$M_{2}=\left\{\hat{{\text{VIM}}_{X_{i}}^{\prime }}|\hat{{\text{VIM}}_{X_{i}}^{\prime }}=0;\;i=1,\ldots ,p\right\},$$
  $$M_{3}=\left\{-\hat{{\text{VIM}}_{X_{i}}^{\prime }}|\hat{{\text{VIM}}_{X_{i}}^{\prime }}<0;\;i=1,\ldots ,p\right\}$$
  and consider the empirical cumulative distribution ${\hat{F}}_{0}$ of *M*.
3. Compute the *p*-value corresponding to the variable importance of variable *X_i_* as $p_{i}=1-{\hat{F}}_{0}\left(\hat{{\text{VIM}}_{X_{i}}^{\prime }}\right)$.

Although the impurity-based importance cannot be used because it is always positive, Janitza *et al.* (2016) also show that positive values are more likely for the classical permutation importance and it is thus not entirely unbiased (see also our results in Section 3.1). They use the holdout importance (described in Section 2.2) as an unbiased alternative. The testing approach is implemented in the vita package (https://cran.r-project.org/package=vita) for the holdout importance and in ranger (Wright and Ziegler, 2017) for the holdout and AIR importance. Because the AIR importance is also symmetric around zero under the null hypothesis, it can be used in place of the holdout importance, since it is computationally faster.

### 2.6 Simulation studies

#### 2.6.1 Bias of importance measures

To study the bias of the standard impurity importance, permutation importance, holdout importance and the new AIR importance under the null hypothesis of no association with the outcome, i.e. y⊥X, we performed simulations based on three scenarios. The ranger package was used for all simulations.

In all simulation scenarios, the sample size was set to 100. In case of classification, the outcome variable was generated as a factor from a binomial distribution with probability equal to 0.5, for regression outcomes from a standard normal distribution and for survival outcomes from an exponential distribution (see Supplementary Material for details). The RF were grown with 50 trees and a terminal node size of 1, 5 and 3 for classification, regression and survival, respectively. The option respect.unordered.factors was set to ‘partition’ to consider all possible 2-partitions for splitting unordered factors, as in the original implementation of RF. All simulations were repeated 10^6^ times. In each simulation run, we computed the four VIMs for all covariates. The covariates in the simulation scenarios were simulated as follows:

***Null Case A: Increasing minor allele frequency***

Ten covariates $X_{1},\ldots ,X_{10}$ with increasing minor allele frequency MAF={0.05,0.10,0.15,0.20,0.25,0.30,0.35,0.40,0.45,0.50} were simulated.

***Null Case B: Increasing numbers of categories***

Ten covariates $X_{1},\ldots ,X_{10}$ with increasing numbers of categories were generated from a uniform distribution in a similar fashion as in Altmann *et al.* (2010), i.e. k={2,3,4,5,6,7,8,10,20,30}, where *k* is the number of categories.

***Null Case C: Mixed-type covariates***

Covariates were simulated according to the following scheme: $B_{0.05},\;B_{0.1},\;B_{0.2},\;B_{0.5}$ are binomial with increasing probabilities equal to P={0.05,0.1,0.2,0.5}, *O*_5_, *O*_10_ are ordered factors with 5 and 10 categories, $N_{5},N_{8},N_{10}$ are nominal factors with 5, 8, and 10 categories, and *C* is a continuous covariate generated from a standard normal distribution. Nominal and ordered factors are generated so that each category has the same frequency.

#### 2.6.2 Power study

To evaluate the combination of the AIR importance with the testing procedure by Janitza *et al.* (2016), we analyzed the type I error and power on two gene expression and one GWAS dataset. The first gene expression dataset is from a study of leukemia (Golub, 1999) and consists of data from 72 leukemia patients: 25 with class *acute myeloid leukemia* and 47 with class *acute lymphoblastic leukemia*, with expression measurements from 7129 genes. The classification task is to predict the class of each patient, based on the gene expression data. The second dataset is from a study of breast cancer (van’t Veer *et al.*, 2002) on 76 breast cancer patients: 33 who developed distant metastases within 5 years (cases) and 43 who continued to be disease-free (controls), with expression measurements from 4948 genes. Two patients (1 case, 1 control) with missing data were excluded. The GWAS dataset is from a study of Alzheimer (Webster *et al.*, 2009), including 364 individuals: 176 Alzheimer patients (cases) and 188 healthy controls, with 380 157 SNPs. The dataset was preprocessed and quality controlled by Webster *et al.* (2009). Missing genotype data was treated as an additional category during analysis.

As done by Janitza *et al.* (2016), we used the design matrix of the original datasets and simulated the outcome with a logit model with given effect size. For the effect sizes β={−1,1,−2,2,−3,3,−4,4}, we used 10 variables each, totaling in 80 effect variables. All other variables were not associated with the outcome. We removed correlation between variables by permutation.

For the gene expression data, we generated 1000 datasets and grew an RF with 5000 trees on each dataset. A total of 500 variables were selected for splitting in each node (mtry = 500). For each variable a *p*-value was estimated by every RF and the null hypothesis of no association between the variable and the outcome was rejected if p≤0.05. For each variable the power is the proportion of rejected hypotheses in all replications. Finally, for each dataset, the power is averaged over all variables with the same absolute value of the effect size. The type I error is the proportion of rejected hypotheses of the unassociated variables. For the GWAS data, we used the same analysis scheme with an mtry value of 50 000. Here, we reduced the number of replications to 100 due to computational restrictions. Further simulations with regression and survival outcomes as well as splitting with maximally selected rank statistics (Wright *et al.*, 2017) are presented in the Supplementary Material.

### 2.7 Real data applications

#### 2.7.1 Identification of splice junctions in the DNA dataset

The DNA dataset (Noordewier *et al.*, 1991) consists of 3186 observations, described by 180 binary indicator variables and the outcome variable has the possible classes *ei*, *ie* or *n*. Each observation represents a 60-residue DNA sequence in a way so that each consecutive triplet of indicator variables encodes one residue. The objective is to recognize and classify the sequences containing a boundary between exons (the parts of the DNA sequence retained after splicing) and introns (the parts of the DNA sequence that are spliced out) or vice versa. All sequences were aligned in a way that the boundary lies between the 30th and 31st residue, i.e. the 90th to 96th indicator variable. Because the biological process of recognition is local, the most important variables should be those describing residues in the vicinity of the boundary.

To show how the new VIM performs on a real dataset, we compared the results obtained from a 10-fold cross validation, repeated 10 times. We compared the AIR importance to the classical permutation importance and the holdout importance. The impurity importance was excluded based on the results of the simulation studies. We grew RF with 5000 trees, grown to purity. The resulting importance values were normalized to be between 0 and 1 for graphical purposes. We also computed the Pearson and Spearman correlation coefficients between the different importance measures.

#### 2.7.2 Computational performance

To compare the computational performance of the VIMs in a realistic setting, we analyzed each of the datasets described in Section 2.6.2 with each importance measure and measured the execution time. Each RF was grown with 5000 trees, 1 computing thread and all other settings default. A 64-bit Linux platform with two 16-core Intel Xeon E5-2698 v3 CPUs was used for the computations.

## 3 Results

### 3.1 Simulation studies

#### 3.1.1 Bias of importance measures

**Null Case A: Increasing minor allele frequency**

**Fig. 1.**
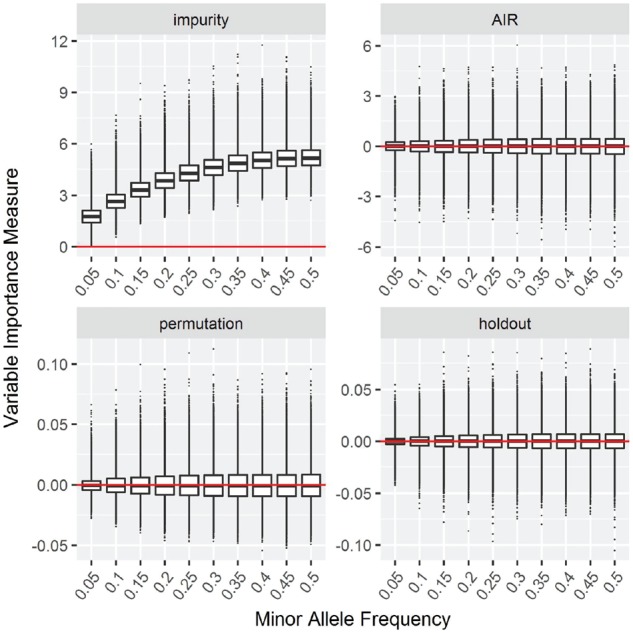
Null Case A: Increasing minor allele frequency. Box plots of simulation replications of four VIMs: impurity, AIR, permutation and holdout importance. Data simulated without association between outcome and covariates. The horizontal line indicates a variable importance of 0

The results for the simulation study with increasing MAF are shown in Figure 1 for classification and in Supplementary Figures S1–S5 for regression, survival and maximally selected rank statistics. In the four panels, the variable importance values of the impurity importance, the proposed AIR importance, the permutation importance and the holdout importance are shown in boxplots of the simulation replications. Since the data are simulated under the null hypothesis of no association between the outcome and the covariates, the expected result for an unbiased importance measure are similar importance values for all covariates.

As previously shown by Boulesteix *et al.* (2012), the impurity importance shows increasing values as the MAF increases. The AIR importance and the holdout importance are both symmetric around zero for all covariates. The regular permutation importance is centered around zero, with positive outliers being more likely. As explained in Janitza *et al.* (2016), this is presumably due to the overlap of out-of-bag observations. For the permutation, holdout and AIR importance, the variance increases slightly towards higher MAF.

***Null Case B: Increasing numbers of categories***

**Fig. 2.**
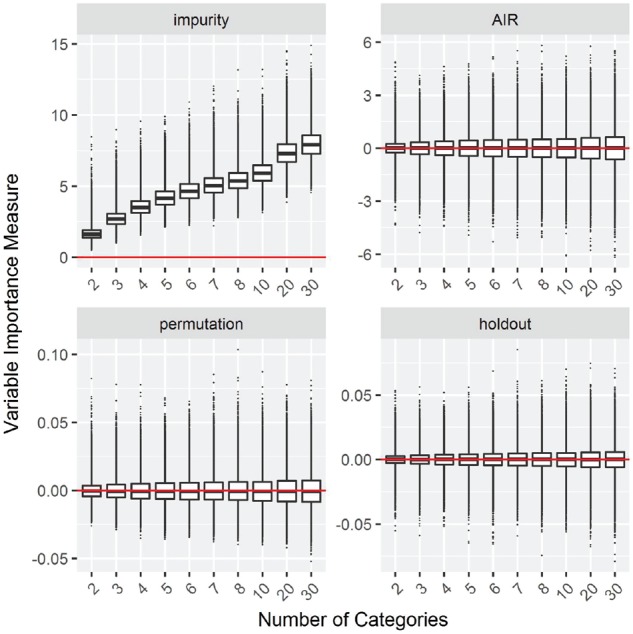
Null Case B: Increasing numbers of categories. Box plots of simulation replications of four VIMs: impurity, AIR, permutation and holdout importance. Data simulated without association between outcome and covariates. The horizontal line indicates a variable importance of 0

The results for the simulation study with an increasing numbers of categories are shown in Figure 2 for classification and in Supplementary Figures S6–S10 for regression, survival and maximally selected rank statistics. As above, the data are simulated under the null hypothesis and the expected result for an unbiased importance measure are similar importance values for all covariates.

The results are similar to the simulation with increasing MAF: The impurity importance is biased in favor of variables with many categories (Strobl *et al.*, 2007) and the other measures are symmetric around zero for all covariates. Again, for the permutation importance positive outliers are more likely, and for the permutation, holdout and AIR importance, the variance increases slightly towards many categories.

***Null Case C:Mixed-type covariates***

**Fig. 3.**
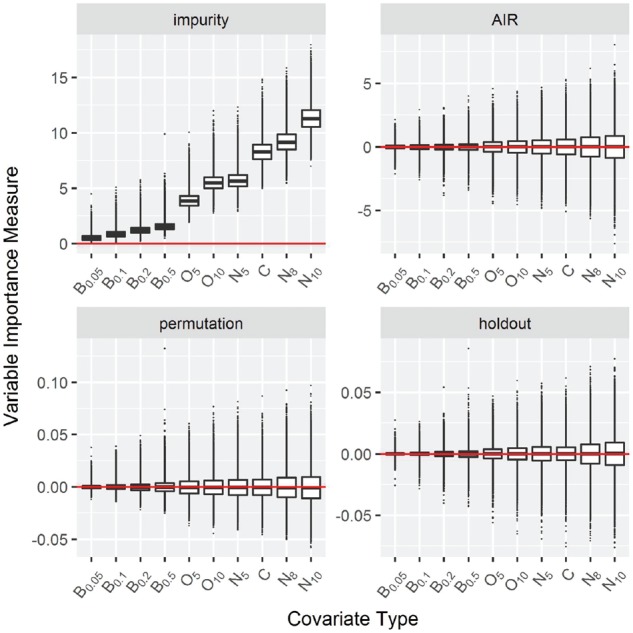
Null Case C: Mixed-type covariates. Box plots of simulation replications of four VIMs: impurity, AIR, permutation and holdout importance. The covariates are described in Section 2.6.1. Data simulated without association between outcome and covariates. The horizontal line indicates a variable importance of 0

In the third simulation scenario, the covariates are of mixed type. As explained in Section 2.6.1, four variables are binomial with different frequencies, two are ordered factors, three are unordered factors and one variable is continuous. The results are shown in Figure 3 for classification and in Supplementary Figures S11–S15 for regression, survival and maximally selected rank statistics. As in the previous simulations, the data are simulated under the null hypothesis and the expected result for an unbiased importance measure are similar importance values for all covariates.

As expected the impurity importance shows increasing values as the number of splitting points increases: Binary variables get a small variable importance, while nominal and continuous variables get the highest scores. In this simulation scenario the variance increases with the number of potential split points for all importance measures.

#### 3.1.2 Power study

**Fig. 4.**
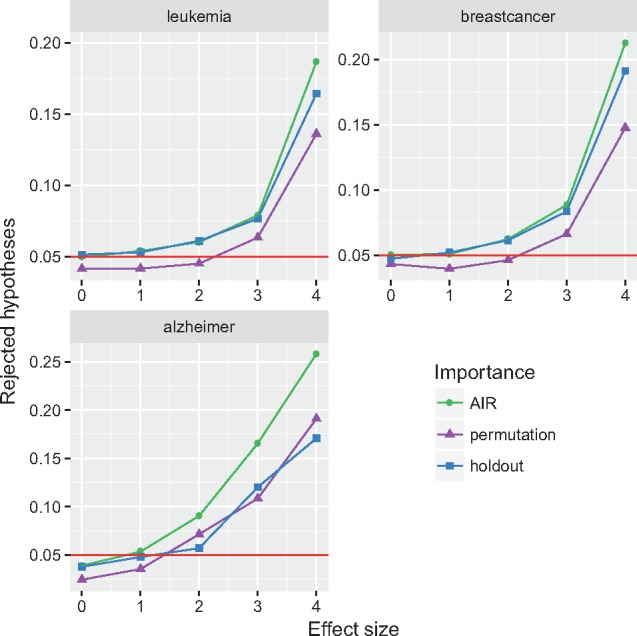
Results of the power study. The panels correspond to the analyzed datasets, the colors and symbols to the importance measures. Average proportion of rejected hypotheses at α=0.05. Results at effect size 0 correspond to the type I error, at effect sizes >0 to statistical power. The horizontal line indicates the nominal level of α=0.05

The results of the power study are shown in Figure 4. Each point corresponds to the average proportion of rejected hypotheses at α=0.05 for a given dataset and importance measure. On the gene expression datasets, the type I errors of all three importance measures were at or below the nominal level. As expected, the power was increasing with the effect size. The test based on the permutation importance showed slightly lower power, compared with the AIR and holdout importance, which were approximately on par. On the GWAS data, the type I errors of all three importance measures were below the nominal level. Here, the power of the test based on the permutation importance was on par with the holdout importance and both were outperformed by the AIR importance. Experiments with the AIR importance showed that with a higher number of trees, the type I errors leveled at the nominal level (Supplementary Fig. S17). Further results for data with regression and survival outcomes are shown in Supplementary Figure S16.

### 3.2 Real data applications

#### 3.2.1 Identification of splice junctions in the DNA dataset

**Fig. 5.**
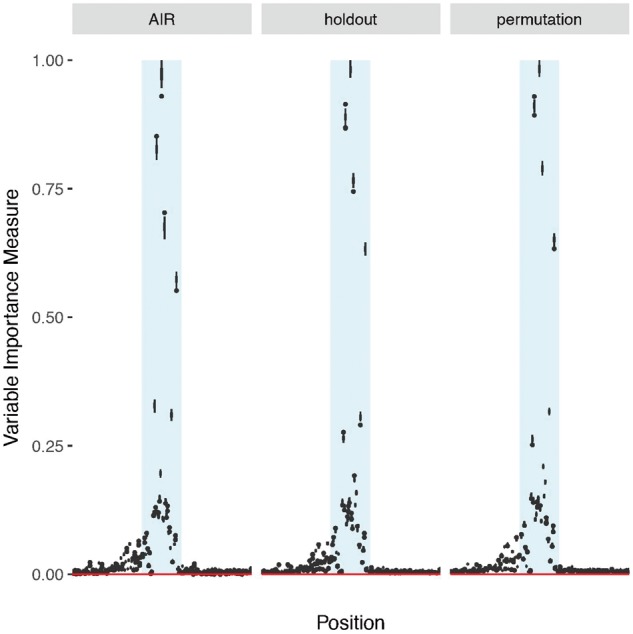
Normalized importance scores for the DNA dataset. From left to right: the AIR, holdout and the permutation importance. All importance measures peak at variables 90–96 (shaded area), corresponding to residues 30–31, i.e. the actual splicing site

The results for the application to the DNA dataset are shown in Figure 5. The panels correspond to the AIR, holdout and permutation importance measures. The estimated importance values, normalized to [0,1], for all positions are shown.

The results show that all the importance measures are consistent with the expectations based on the dataset structures. One can notice a maximum around positions 90–96 (shaded area), corresponding to the actual cleavage site location. The values obtained for the AIR importance were very close to the classical and the holdout permutation importance with median Pearson (Spearman) correlation coefficients equal to 0.995 (0.964) and 0.996 (0.956), respectively.

#### 3.2.2 Computational performance

The results of the runtime comparison are shown in Table 1. On all datasets, the impurity importance was the fastest with runtimes between 4 s for the gene expression data and about 7 min for the GWAS data, closely followed by the AIR importance. The permutation importance was approximately 10 times slower for the gene expression data and about 50 times slower for the GWAS data. The holdout importance was slowest with a 20–30-fold increase for the gene expression data and more than a 100-fold increase for the GWAS data, compared with the impurity importance. Further results for data with regression and survival outcomes are shown in Supplementary Table S1.

**Table 1.** Runtime (s) for growing a RF and computing a VIM on two gene expression and one GWAS dataset

Importance	Gene expr.		GWAS
	Leukemia	Breast cancer	Alzheimer
Impurity	3.7	4.2	439.3
Permutation	46.8	38.4	21 511.5
Holdout	112.5	89.1	48 772.5
AIR	3.9	5.5	535.6

*Note*: The RF is grown with 5000 trees using one computing thread.

## 4 Discussion

We have shown that the corrected impurity importance measure (AIR) is almost as fast as the standard impurity importance and much faster than the permutation importance, and is simultaneously unbiased with regard to the number of categories or MAF in SNPs. With the AIR importance it is now possible to compute reliable importance estimates without the extra computing cost of permutations or pseudo variables. Further, we have shown that combined with the testing procedure of Janitza *et al.* (2016), our method can be used to estimate *p*-values, keeping the significance level and with high statistical power. We have shown that all results and conclusions apply also to regression forests with the sum of squares as impurity importance, survival forests with the log-rank test statistic (Ishwaran *et al.*, 2008) and to RFs with maximally selected rank statistics (Wright *et al.*, 2017).

As explained in Section 2.4, the tree growing procedure is slightly altered when the AIR importance is computed. Because a fraction of the splits are uninformative, this could lead to a loss of prediction accuracy of the RF, in particular if the mtry value is low. Consequently, we recommend to grow a separate RF for prediction when the AIR importance is used and the ranger package issues a warning when prediction is performed with a forest grown with the AIR importance. Alternatively, the user may decide to build a new RF using the *p*-values as sampling weights for the variables, in a similar fashion as in ‘Guided RF’ (Deng, 2013). Further, based on our results on the GWAS data, we recommend to increase the number of trees when computing VIMs for extremely high dimensional data. It should also be noted that all VIMs considered here may be influenced by correlations between variables in the model (Nicodemus and Malley, 2009; Strobl *et al.*, 2008). This may help finding genetic markers, e.g. in GWAS, but could also lead to spurious signals (Nicodemus *et al.*, 2010). Further simulations are required to study the performance of the AIR importance for correlated variables.

Although the AIR importance can be applied to datasets of any size, the testing procedure of Janitza *et al.* (2016) is limited to high dimensional data with many uninformative variables because it relies on negative importance values. In other situations, the AIR importance can be combined with a permutation testing approach (Altmann *et al.*, 2010). As an alternative, some uninformative variables could be added to the dataset or permuted samples could be used, similar to the AIR importance. A different approach to compute unbiased variable importance estimates for smaller datasets are conditional inference forests (Hothorn *et al.*, 2006) with the permutation importance or conditional permutation importance (Strobl *et al.*, 2008).

In summary, we proposed a unified framework to compute a fast VIM for RF, which does not suffer from bias due to different MAF or numbers of categories in the variables. The corrected impurity importance measure (AIR) outperforms previous approaches in terms of computational performance and statistical power. We recommend to use it as a general replacement for the impurity and permutation importances, in particular for high dimensional data from the life sciences.

## Funding

This work was partly funded by the Deutsche Forschungsgemeinschaft [CRU303 Z2, FOR2488 P7 and KO2250/5-1] to I.R.K.

*Conflict of Interest*: none declared.

## Supplementary Material

Supplementary Data
