# Supplementary material for: The revival of the Gini importance?
Source: Bioinformatics. 2018 May 10;34(21):3711–8. doi: 10.1093/bioinformatics/bty373 (PMC6198850; doi:10.1093/bioinformatics/bty373)
Supplement: Supplementary Data [file bty373_supplement_nembrini.pdf]

Supplementary material for ”The revival of the Gini importance?”

Stefano Nembrini, Inke R. Konig and Marvin N. Wright

April 4, 2018

1 Null case simulations for regression, survival and maximally selected rank statistics

Plots for null cases A, B and C for regression, survival and maximally selected rank statistics. For regression, the variable importance is computed as the sum of squares and for survival as the sum of logrank statistics. For maximally selected rank statistics, the importance is computed as the sum of maximally selected rank statistics.

The classification outcome was simulated from a binomial distribution with probability equal to 0.5, the regression outcome from a standard normal distribution. The survival outcome was simulated by the minimum of a survival and censoring time, both assumed to be exponentially distributed with $\lambda = 0.5$ and $\lambda = 0.1$, respectively.

Simulations were replicated 1000 times and forests grown with 50 trees. For classification with maximally selected rank statistics, the outcome was coded as 0 and 1 and regression forests were grown to estimate probabilities.

1.1 Null Case A

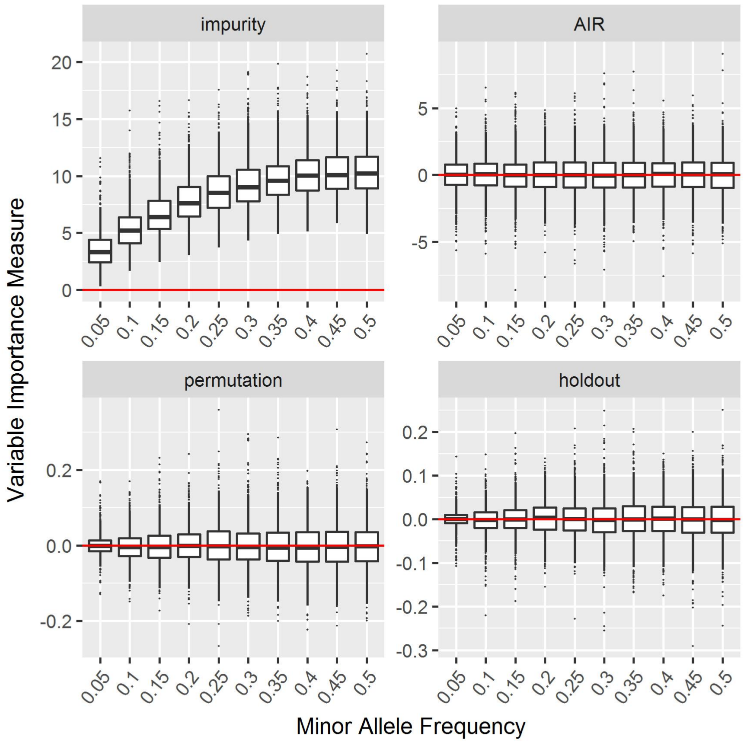

Figure S1: Null Case A - Regression: Increasing minor allele frequency. Boxplots of simulation replications of four variable importance measures: Impurity, AIR, permutation and holdout importance. Data simulated without association between outcome and covariates. The red line indicates a variable importance of 0.

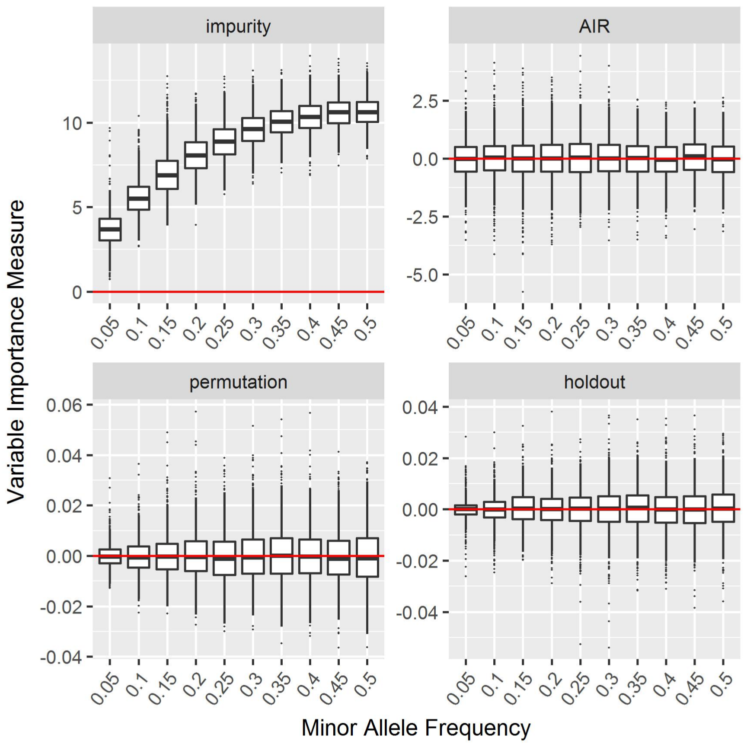

Figure S2: Null Case A - Survival: Increasing minor allele frequency. Boxplots of simulation replications of four variable importance measures: Impurity, AIR, permutation and holdout importance. Data simulated without association between outcome and covariates. The red line indicates a variable importance of 0.

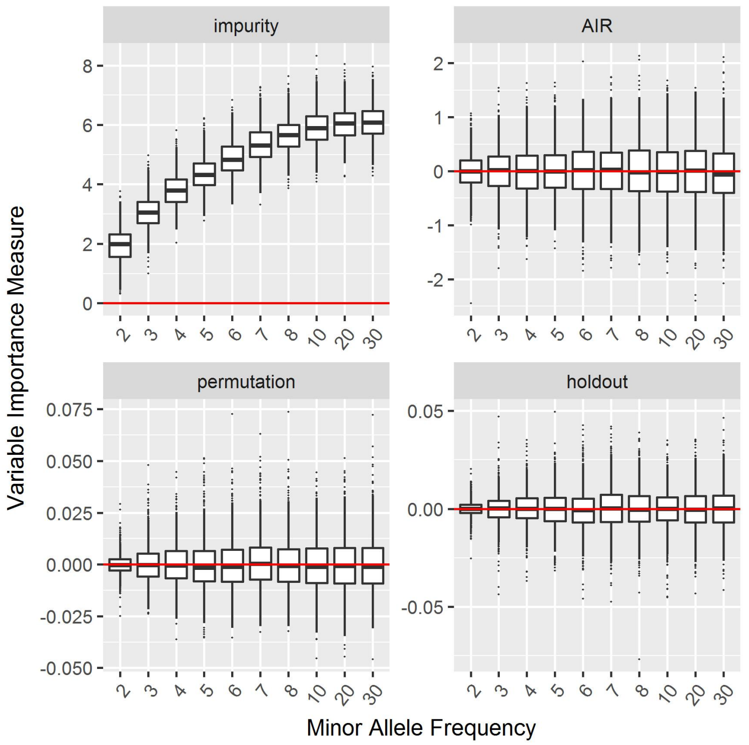

Figure S3: Null Case A - Maximally Selected Rank Statistics - Classification: Increasing minor allele frequency. Boxplots of simulation replications of four variable importance measures: Impurity, AIR, permutation and holdout importance. Data simulated without association between outcome and covariates. The red line indicates a variable importance of 0.

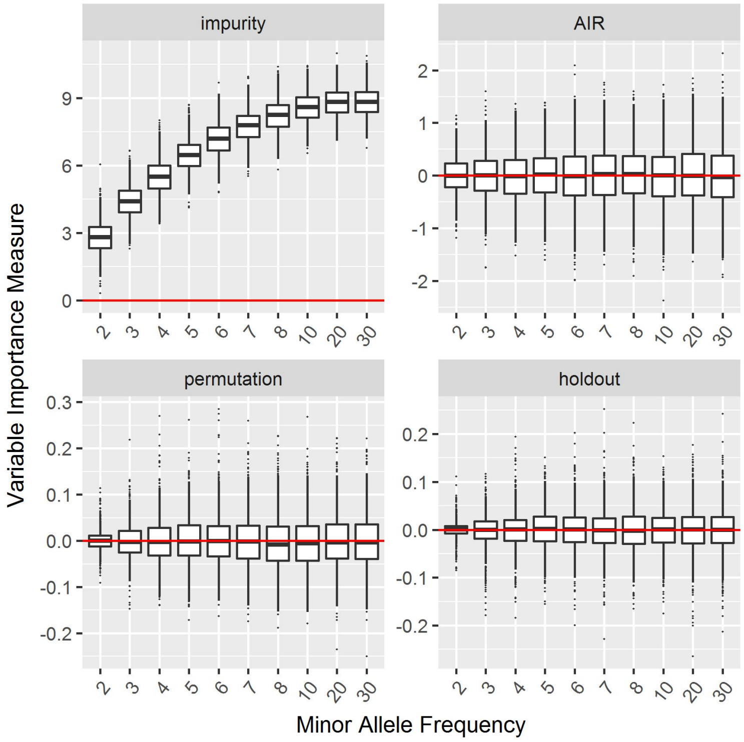

Figure S4: Null Case A - Maximally Selected Rank Statistics - Regression: Increasing minor allele frequency. Boxplots of simulation replications of four variable importance measures: Impurity, AIR, permutation and holdout importance. Data simulated without association between outcome and covariates. The red line indicates a variable importance of 0.

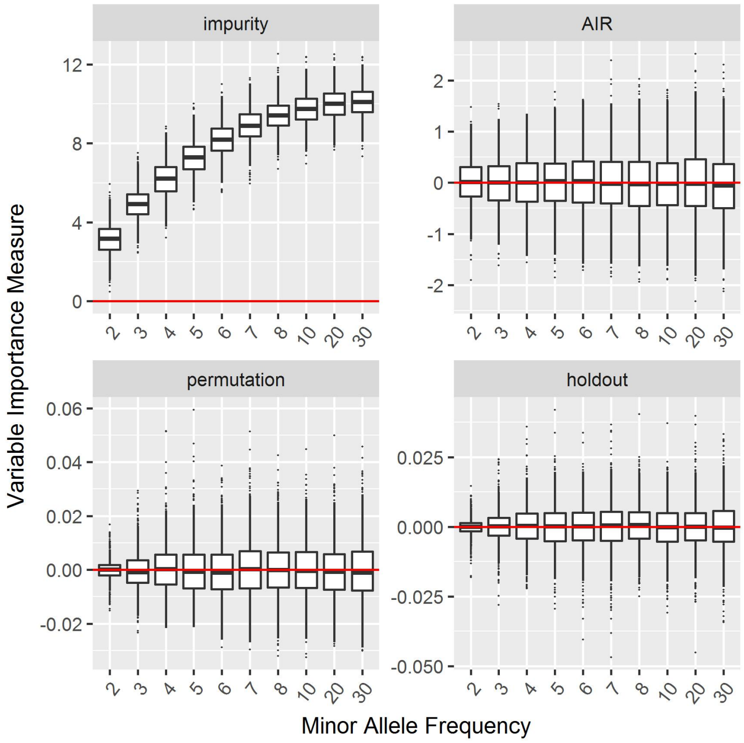

Figure S5: Null Case A - Maximally Selected Rank Statistics - Survival: Increasing minor allele frequency. Boxplots of simulation replications of four variable importance measures: Impurity, AIR, permutation and holdout importance. Data simulated without association between outcome and covariates. The red line indicates a variable importance of 0.

1.2 Null Case B

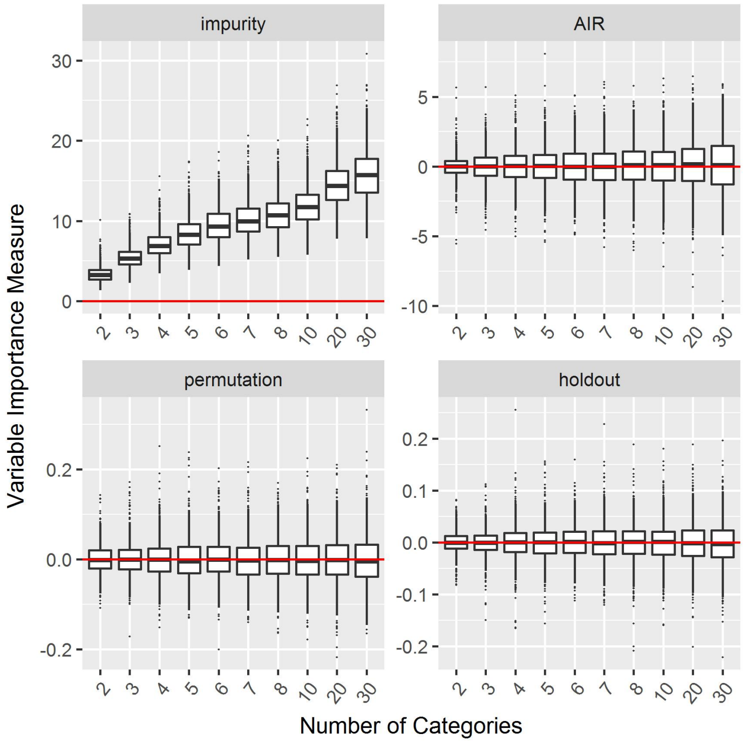

Figure S6: Null Case B - Regression: Number of Categories. Boxplots of simulation replications of four variable importance measures: Impurity, AIR, permutation and holdout importance. Data simulated without association between outcome and covariates. The red line indicates a variable importance of 0.

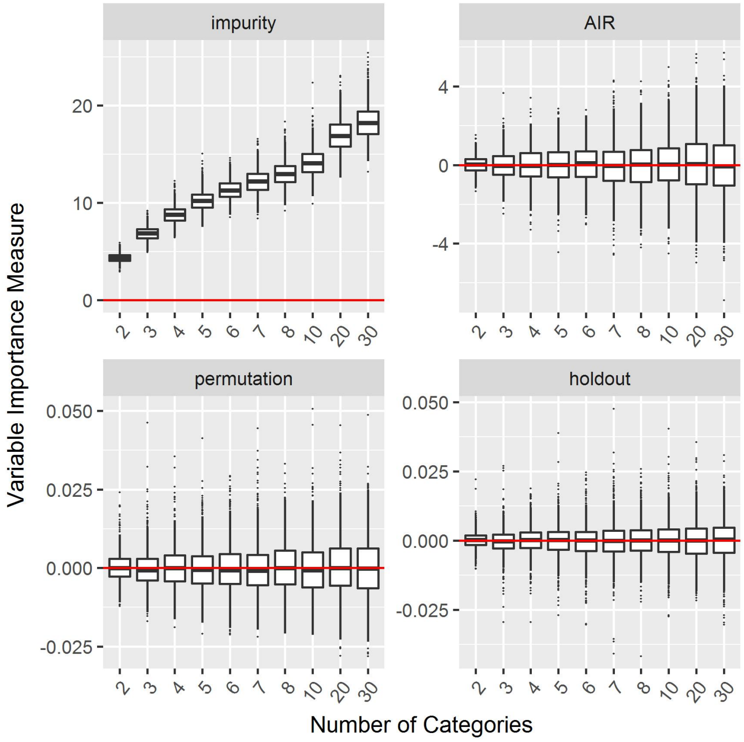

Figure S7: Null Case B - Survival: Number of Categories. Boxplots of simulation replications of four variable importance measures: Impurity, AIR, permutation and holdout importance. Data simulated without association between outcome and covariates. The red line indicates a variable importance of 0.

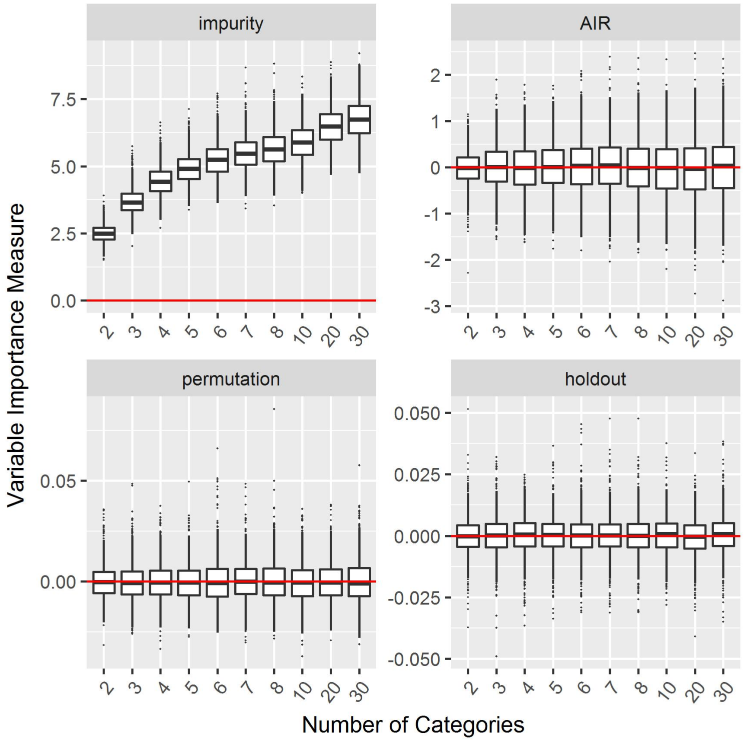

Figure S8: Null Case B - Maximally Selected Rank Statistics - Classification: Number of Categories. Boxplots of simulation replications of four variable importance measures: Impurity, AIR, permutation and holdout importance. Data simulated without association between outcome and covariates. The red line indicates a variable importance of 0.

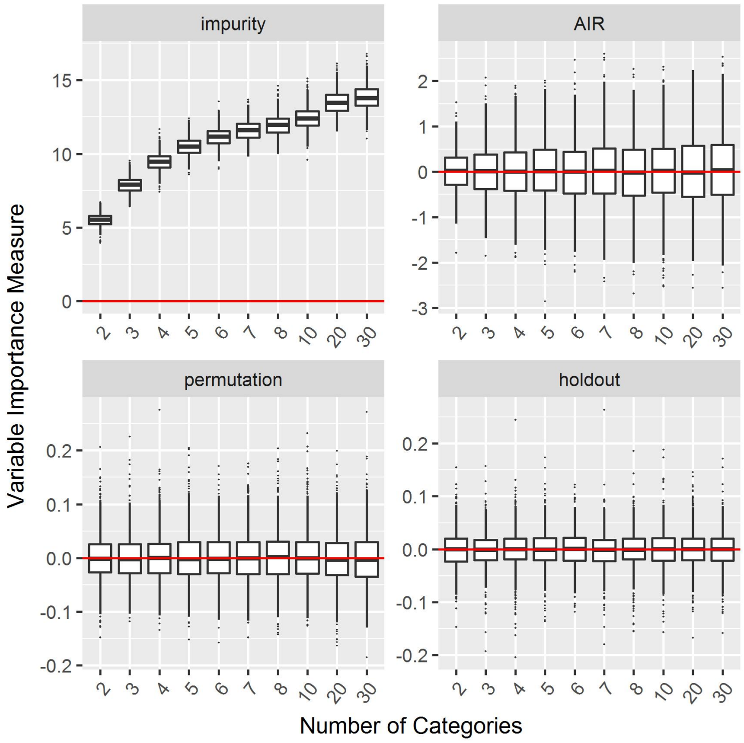

Figure S9: Null Case B - Maximally Selected Rank Statistics - Regression: Number of Categories. Boxplots of simulation replications of four variable importance measures: Impurity, AIR, permutation and holdout importance. Data simulated without association between outcome and covariates. The red line indicates a variable importance of 0.

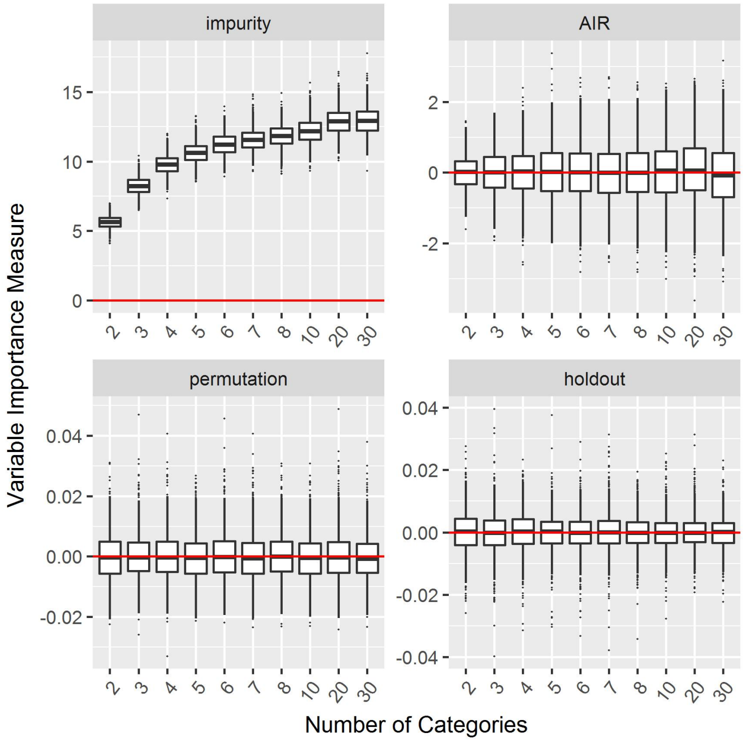

Figure S10: Null Case B - Maximally Selected Rank Statistics - Survival: Number of Categories. Boxplots of simulation replications of four variable importance measures: Impurity, AIR, permutation and holdout importance. Data simulated without association between outcome and covariates. The red line indicates a variable importance of 0.

1.3 Null Case C

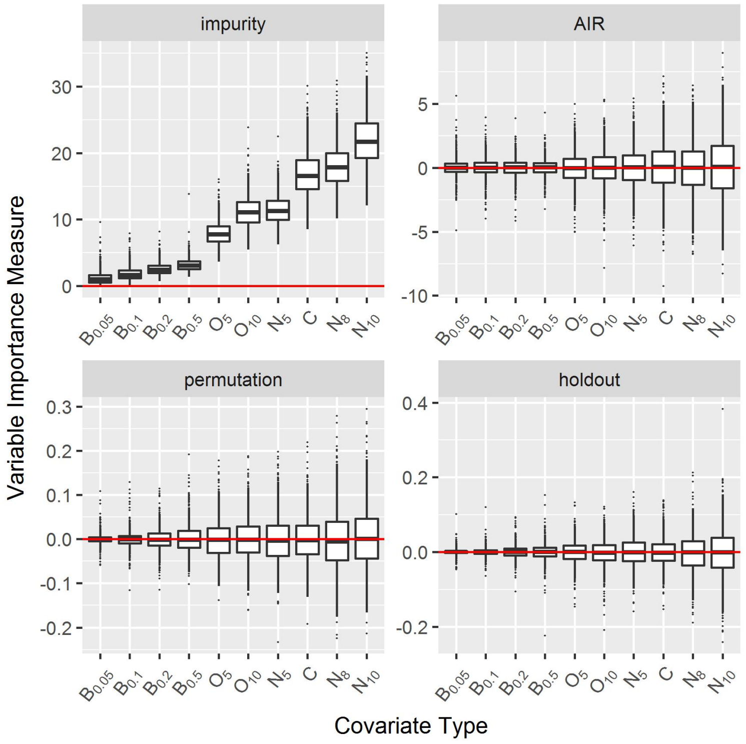

Figure S11: Null Case C - Regression: Covariate Type. Boxplots of simulation replications of four variable importance measures: Impurity, AIR, permutation and holdout importance. Data simulated without association between outcome and covariates. The red line indicates a variable importance of 0.

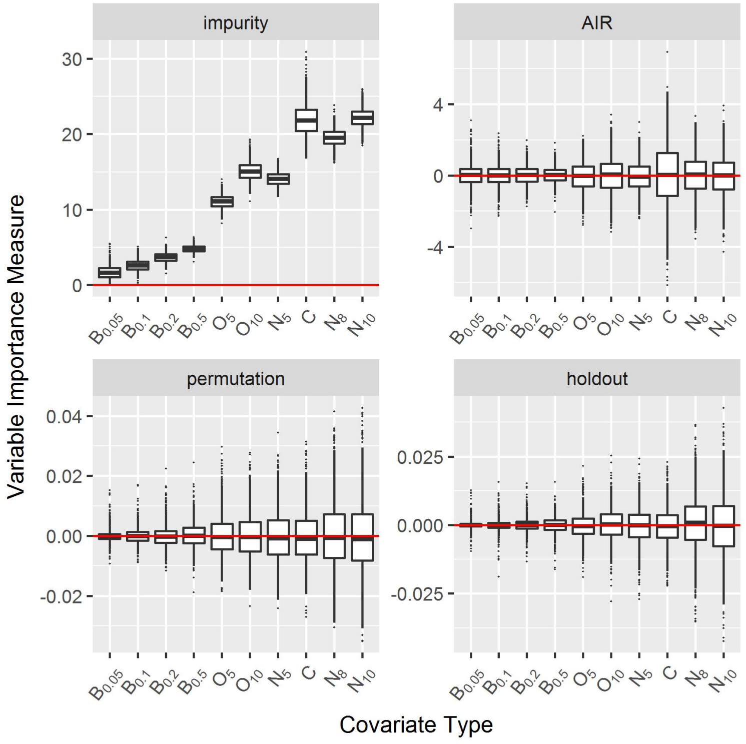

Figure S12: Null Case C - Survival: Covariate Type. Boxplots of simulation replications of four variable importance measures: Impurity, AIR, permutation and holdout importance. Data simulated without association between outcome and covariates. The red line indicates a variable importance of 0.

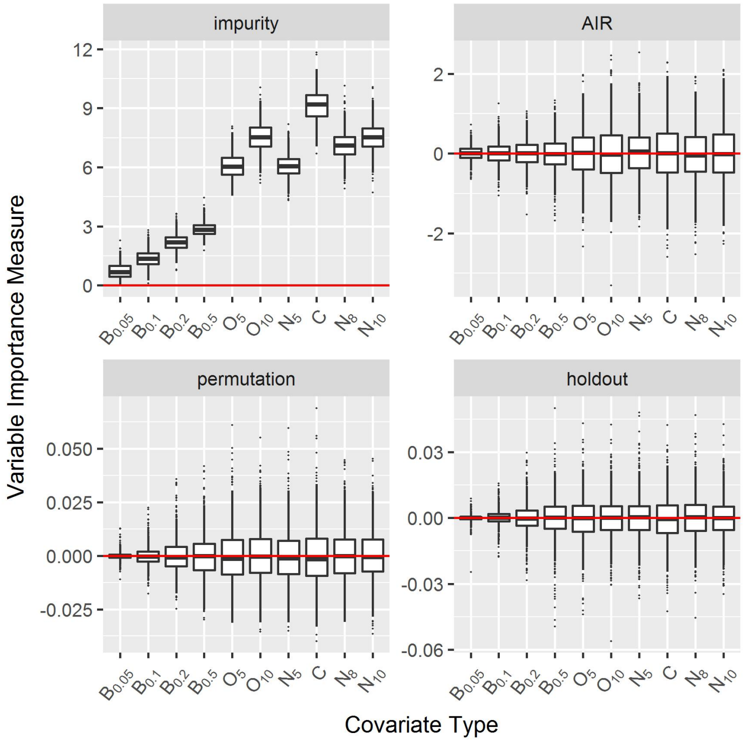

Figure S13: Null Case C - Maximally Selected Rank Statistics - Classification: Covariate Type. Boxplots of simulation replications of four variable importance measures: Impurity, AIR, permutation and holdout importance. Data simulated without association between outcome and covariates. The red line indicates a variable importance of 0.

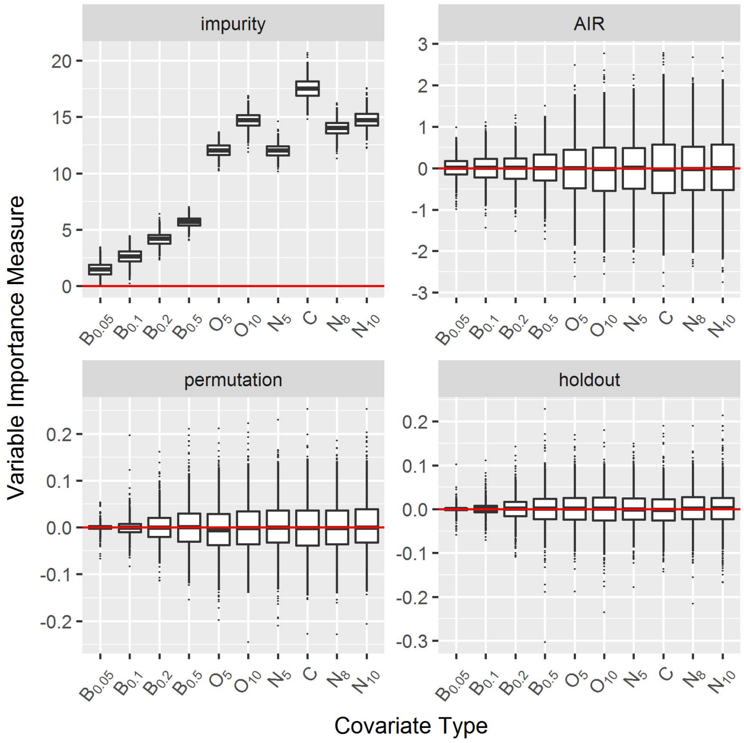

Figure S14: Null Case C - Maximally Selected Rank Statistics - Regression: Covariate Type. Boxplots of simulation replications of four variable importance measures: Impurity, AIR, permutation and holdout importance. Data simulated without association between outcome and covariates. The red line indicates a variable importance of 0.

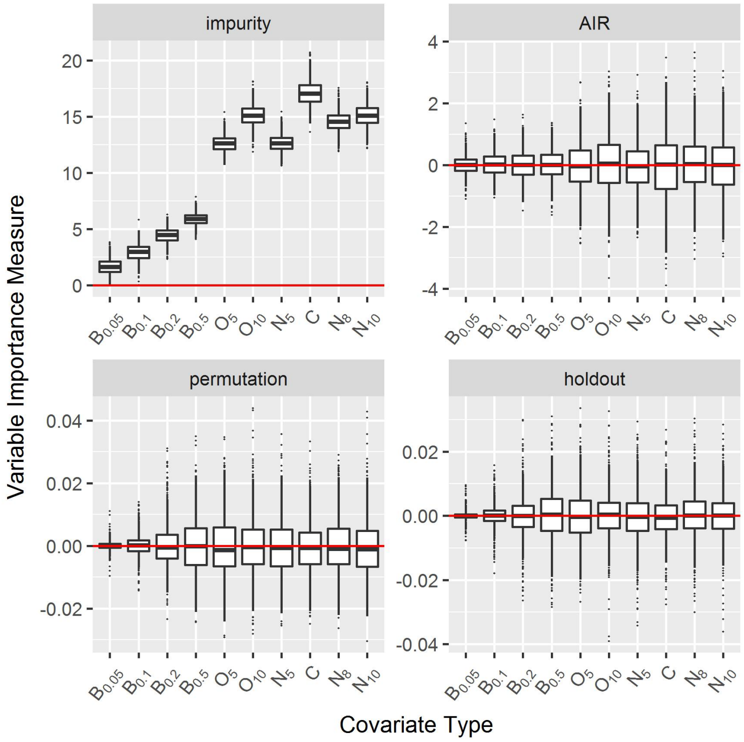

Figure S15: Null Case C - Maximally Selected Rank Statistics - Survival: Covariate Type. Boxplots of simulation replications of four variable importance measures: Impurity, AIR, permutation and holdout importance. Data simulated without association between outcome and covariates. The red line indicates a variable importance of 0.

2 Power study for regression and survival data

The `mouse` dataset (Horvath, 2011) consists of data from 135 female mice with expression measurements from 3600 genes. The regression task is to predict the body weight, based on the genetic data. We excluded mice with missing phenotype data (n=3), excluded genes with more than 5% missing data (n=94) and imputed the remaining missing data with the column-wise median. The final dataset consists of 132 mice and 3506 genes.

The `aml` dataset (Bullinger *et al.*, 2004) is a survival dataset with data from 6283 genes, measured in 116 adults with acute myeloid leukemia (AML). The data is available at the GEO data base with accession number *GSE425*. No phenotype data was missing. Again, we excluded genes with more than 5% missing data (n=6283) and imputed the remaining missing data with the column-wise median. The final dataset consists of 116 patients and 3692 genes.

In the `gaw16` dataset (Amos *et al.*, 2009), the task is to predict rheumatoid arthritis affection status based on SNP data. The analyzed dataset includes 2062 individuals (868 cases and 1194 controls) with genotypes of 3973 SNPs from the HLA region on chromosome 6.

For all datasets, 5000 trees, an `mtry` value of 500 and 100 replications were used. All other settings as described in the paper.

The results of the power study are shown in Figure S16. Runtimes are shown in Table S1. To compare the runtime of the AIR importance with the original approach of Sandri and Zuccolotto (2008), it is added to Table S1.

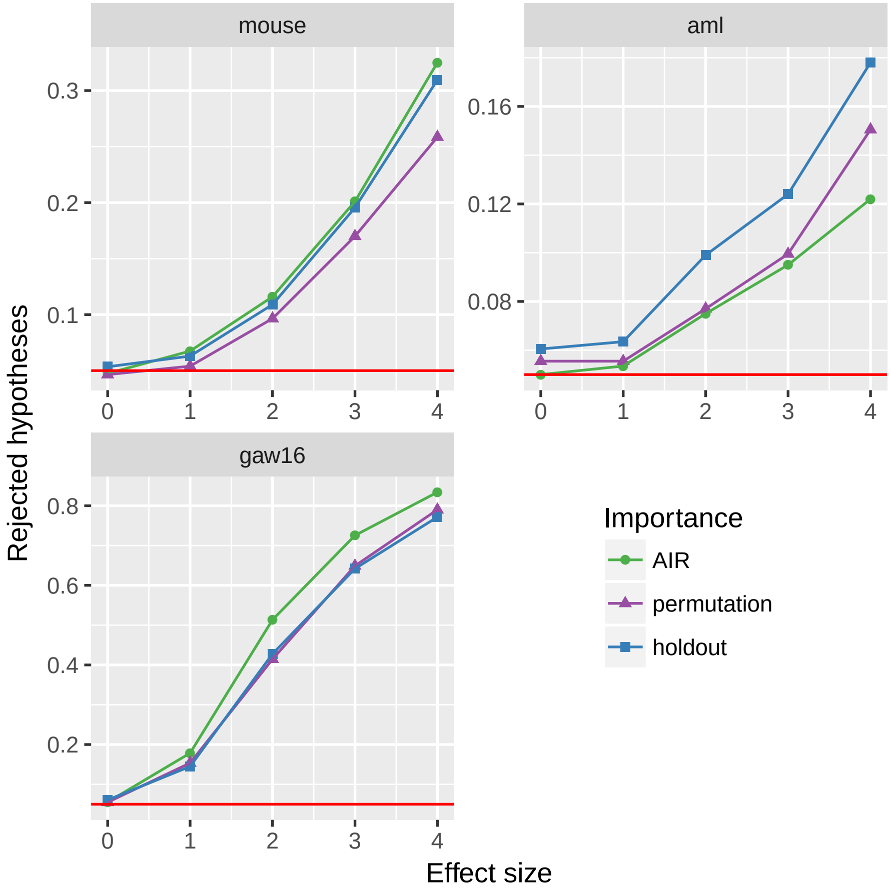

Figure S16: Results of the power study on additional datasets. The panels correspond to the analyzed datasets, the colors and symbols to the importance measures. Average proportion of rejected hypotheses at $\alpha = 0.05$. Results at effect size 0 correspond to the type I error, at effect sizes >0 to statistical power. The red line indicates the nominal level of $\alpha = 0.05$.

Table S1: Extended Table 1. Runtime for growing a random forest and computing a variable importance measure. The random forest is grown with 5000 trees using 1 computing thread. The approach of Sandri and Zuccolotto (2008, S&Z), a regression dataset (Mouse), a survival dataset (AML) and a classification dataset (GAW16) are added. †Estimated with fewer trees.

Importance	Runtime (seconds)					
	Leukemia	Breast cancer	Alzheimer	Mouse	AML	GAW16
Impurity	3.7	4.2	439.3	12.0	79.4	23.8
Permutation	46.8	38.4	21 511.5	83.7	443.2	2011.1
Holdout	112.5	89.1	48 772.5	163.3	1168.4	4632.2
AIR	3.9	5.5	535.6	12.3	79.6	27.0
S&Z (R=1)	5.8	8.1	10 382.6	16.5	118.3	45.6
S&Z (R=100)	503.9	603.4	936 883.7†	1103.9	11 822.9	4490.9

3 Convergence of type I error

The type I error was computed for the AIR importance on the `alzheimer` dataset for an increasing number of trees. All others settings as in the power study.

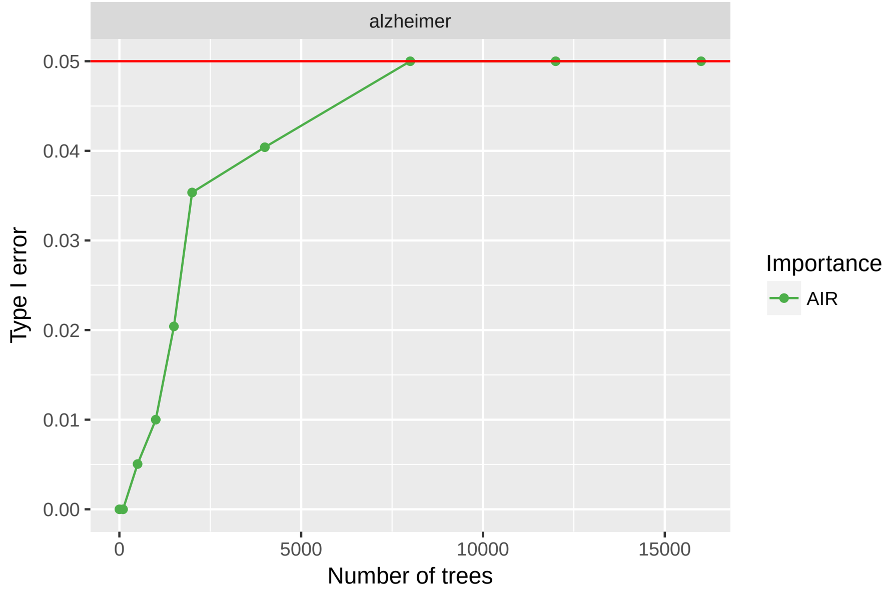

Figure S17: Convergence of the type I error of the AIR importance on the `alzheimer` dataset. The red line indicates the nominal level of $\alpha = 0.05$. Other settings unchanged.

4 Importance for the null case with real data

Plots for the null case on the Leukemia, Breast cancer and Alzheimer data. For Leukemia and Breast cancer, an RF with 5000 trees and $mtry = 500$ was grown, for Alzheimer with 50000 trees and $mtry = 100\,000$. The outcome was permuted before analysis. Each plot shows a histogram of the importance values of all variables in the datasets.

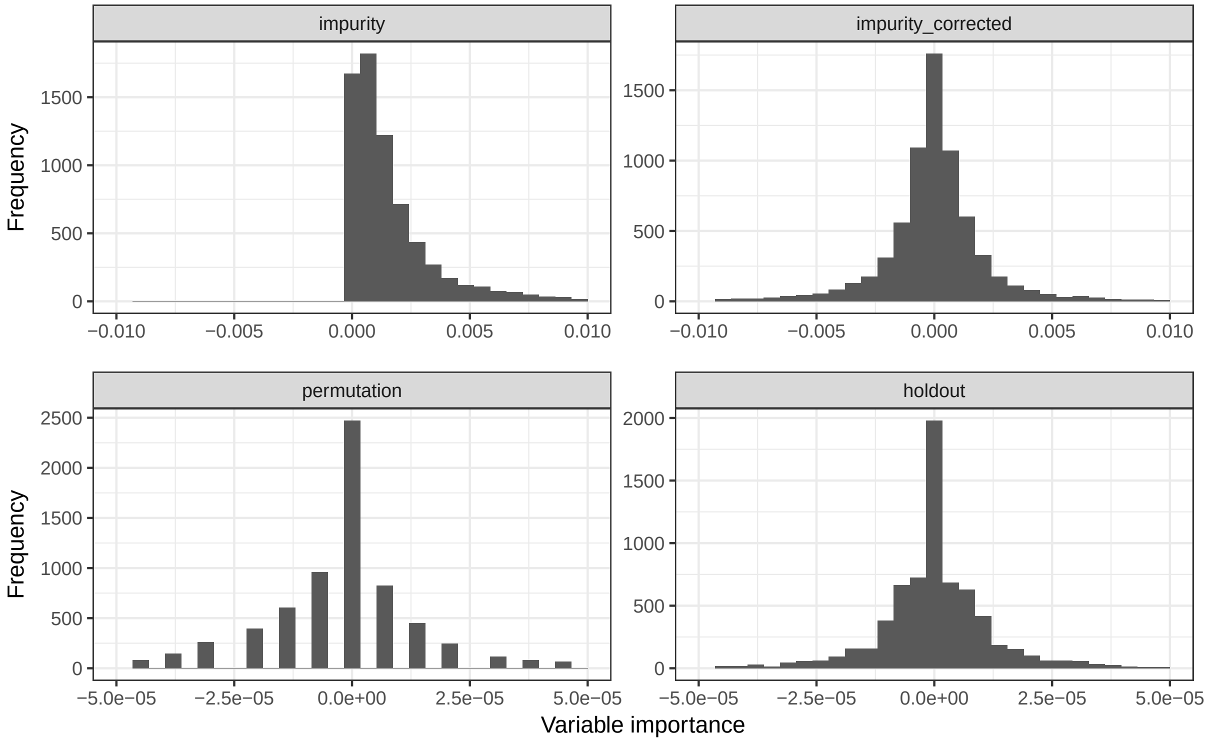

Figure S18: Null importance for Leukemia data: Histograms of four variable importance measures: Impurity, AIR, permutation and holdout importance. Data simulated without association between outcome and covariates.

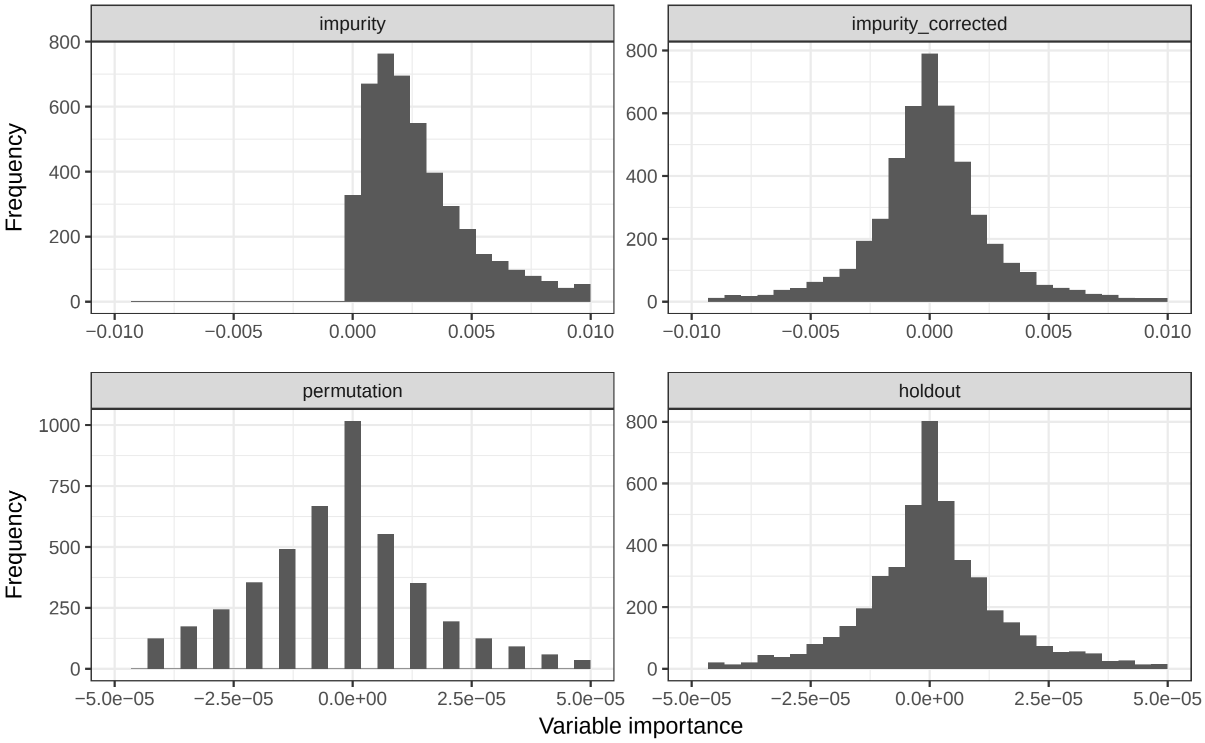

Figure S19: Null importance for Breast cancer data: Histograms of four variable importance measures: Impurity, AIR, permutation and holdout importance. Data simulated without association between outcome and covariates.

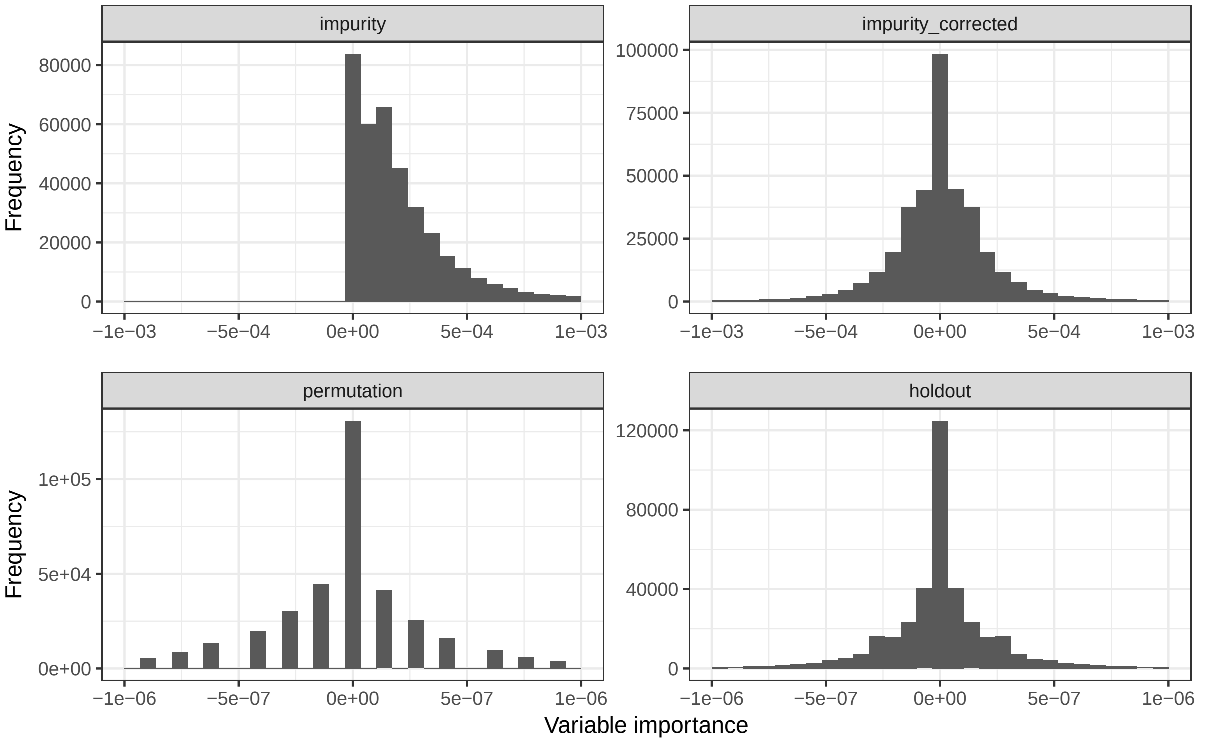

Figure S20: Null importance for Alzheimer data: Histograms of four variable importance measures: Impurity, AIR, permutation and holdout importance. Data simulated without association between outcome and covariates.

Acknowledgements

This work is based on data that was gathered with the support of grants from the National Institutes of Health (NO1-AR-2-2263 and RO1-AR-44422), and the National Arthritis Foundation.

References

Amos, C. I. *et al.* (2009). Data for genetic analysis workshop 16 problem 1, association analysis of rheumatoid arthritis data. *BMC Proc*, **3**, S2.

Bullinger, L. *et al.* (2004). Use of gene-expression profiling to identify prognostic subclasses in adult acute myeloid leukemia. *N Engl J Med*, **350**, 1605–1616.

Horvath, S. (2011). *Weighted Network Analysis: Applications in Genomics and Systems Biology*. Springer, New York.
